# From here to Utopia: Theories of Change in Nonideal Animal Ethics

**DOI:** 10.1007/s10806-022-09894-3

**Published:** 2022-11-03

**Authors:** Nico Dario Müller

**Affiliations:** Philosophical Seminar (Department of Arts, Media, Philosophy), University of Basel, Steinengraben 5, 4051 Basel, Switzerland

**Keywords:** Animal ethics, Nonideal theory, Theory of change, Utopia, Political turn

## Abstract

Animal ethics has often been criticized for an overreliance on “ideal” or even “utopian” theorizing. In this article, I recognize this problem, but argue that the “nonideal theory” which critics have offered in response is still insufficient to make animal ethics action-guiding. I argue that in order for animal ethics to be action-guiding, it must consider *agent-centered theories of change* detailing how an ideally just human-animal coexistence can and should be brought about. I lay out desiderata that such a theory of change should suffice so as to be helpful in guiding action. Specifically, a theory of change should determine (1) who needs to do what in order for ideal justice to be achieved in the long run, (2) who should be expected to refuse compliance and how they should be moved to comply, and (3) why specific intermediate steps are necessary. I show how previous “nonideal” contributions, though helpful in other ways, are insufficiently determinate on these points and I sketch a (still somewhat utopian) theory of change for one specific context. This brings animal ethics a crucial step closer to being action-guiding in the real world.

## Introduction

Animal ethics is supposed to be action-guiding in the real world. But it can only be action-guiding if it is adequately responsive to facts, including facts about individuals, communities, and societies. For example, even if we knew conclusively that all sentient animals have a moral right not to be harmed, and that death is a grave harm to them—two classic issues of contention in animal ethics—it is quite unclear what this view demands of an agent in a particular society in which a vast majority of people are complicit in the slaughter industry. Surely, to personally stop eating meat is not all these principles demand of individuals, if they demand it at all (since an individual’s abstinence is likely inconsequential, Fischer, 2018). But what they demand instead, or in addition, is by no means obvious. This is a problem for any well-meaning decisionmaker or activist who looks to animal ethics for guidance.

In recent years, animal ethics has been criticized for this lack of action-guidance. The criticism is directed particularly against theorists of great influence in the twentieth century such as Singer (2002), Regan (2004), and Francione (1995), and is sometimes couched in terms of the Rawlsian distinction between ideal and nonideal theory. The claim that animal ethics focuses too much on ideal theory and needs to be more “nonideal”, “realistic”, or “pragmatic” (the latter two understood in an everyday sense, not a technical philosophical sense) has been made by philosophers (Garner, 2013; Cochrane et al., 2018; Eskens, 2021; Thompson, 2021), psychologists (Kasperbauer, 2018), legal scholars (Posner, 2004), and activists (Tuider, 2016; Leenaert, 2017). An emphasis on “pragmatism” is also taken to be characteristic of the “political turn” in animal ethics (Milligan, 2015, 156; Ahlhaus & Niesen, 2015, 19). Thus, concerns about the capacity of animal ethics to guide action are prominent in the debate. Though none of the above authors claim that theorizing about fundamental moral principles is *superfluous*, they argue that this type of theorizing needs to be supplemented by nonideal theory, which considers more thoroughly the actual conditions under which ideals of justice are to be realized.

I want to show that, in order to be action-guiding, animal ethics needs agent-centered theories of change, by which I mean accounts of how an initially noncompliant society comes to comply with principles of justice through the efforts of agents. As long as we put off the challenging task of devising such accounts, nonideal animal ethics will continue to be insufficiently action-guiding.

In what follows, I first develop the notion of a theory of change in the context of Rawlsian nonideal theory (Sect. 2). I then argue that such a theory of change should meet three desiderata—it should determine (1) who needs to do what in order for justice to be achieved, (2) who should be expected to be unable or unwilling to comply and how they should be enabled and moved to comply, and (3) why each intermediate step of the theory of change is required on the path to justice (Sect. 3). I then discuss three kinds of nonideal theorizing that dominate ethical debates on animal protection—critique, compromise, and tactics—arguing that each fails to provide a theory of change determinate enough to meet the desiderata (Sect. 4). I conclude by imagining a theory of change focusing on one particular ideal theory, *Zoopolis* (Donaldson and Kymlicka 2011), and one particular nation state, Switzerland (Sect. 5). The theory of change still falls short of being fully realistic, but I argue that a naive theory of change is better than none, and that it can be built upon.

## Nonideal Theory and Theories of Change

The ideal-nonideal distinction originates with Rawls. On Rawls’s conception, ideal theory determines the principles of justice under the assumption of full compliance and favourable circumstances (Rawls 1999, 216). It thus describes a “Realistic Utopia” (Rawls, 1999, 11). Ideal theory may be utopian in its assumptions about contingent circumstances and people’s willingness to comply, but it should not be utopian in any other respects, particularly when it comes to its assumptions about the capabilities of human beings.

Nonideal theory, by contrast, determines how the principles of justice should be implemented in the actual world, where not everybody complies and circumstances are not always favourable (Rawls, 2001, 89). For example, nonideal theory deals with the question how liberal democracies should act towards regimes of states that are not well-ordered according to ideal theory (Rawls, 2001, 90), or how a particular state should move away from an unjust policy of killing prisoners of war (Rawls, 1999, 218).

On Rawls’s conception, ideal theory is a necessary prerequisite of nonideal theory. Ideal theory determines the long-term goal, nonideal theory then determines the right steps towards it. Nonideal theory therefore does not merely *evaluate* or *compare* different real-world institutional arrangements in terms of their conformity to principles of justice. Rather, it is essentially *transitional*, in the sense of investigating what a process of change towards ideal justice could and should look like (Simmons, 2010, 22).

Due to its essentially transitional character, nonideal theory is implicitly tied to the idea of a *theory of change* (TOC)—a notion Rawls did not use, but might as well have used. By a TOC, I mean an account of how an initially noncompliant society comes to comply with principles of justice. Specifically, Rawls focuses on the efforts of *agents.* This contrasts with non-agent-centered theories by empirical scholars of transition, who often prefer to describe transition in terms of causal chains, policy windows, dimensions of influence, or in yet other terms (Gready & Robins, 2020, 284–85). While considering transition in these terms can be fruitful and important for the purposes of description and explanation, focusing on agents fits the prescriptive aim of Rawls’s philosophy. He focuses on what agents need to do because those agents are the addressees of his prescriptions.

An example of a TOC in Rawls’s work is the aforementioned fictional account of a state killing prisoners of war. The transition Rawls envisions is that the state transitions to keeping prisoners of war as slaves by a self-interested decision of the ruling elite, since slaves are more profitable than dead enemy soldiers. Afterwards, the state transitions to a policy of prisoner-of-war exchange with its adversaries, since receiving one’s own prisoners back is yet more profitable than slavery (Rawls, 1999, 218).

Rawls’s treatment of non-well-ordered regimes similarly suggests a certain TOC. Here, we start out with a world community in which not all regimes are well-ordered according to ideal theory—the liberties of their citizens are too restricted, resource inequalities too great. Well-ordered peoples then form intergovernmental institutions to express their critical opinions about non-well-ordered regimes, exerting symbolic pressure. They reinforce this with economic pressure by denying non-well-ordered regimes certain forms of cooperation and assistance. This gives these regimes an interest in becoming well-ordered (Rawls, 2001, 93).

Rawls points out that this is only a general outline for how justice should be achieved: “How to bring all societies to this goal is a question of foreign policy; it calls for political wisdom, and success depends in part on luck” (Rawls, 2001, 93). This suggests that developing transition plans is a collaborative effort. Philosophers should spell out the ideal and determine what kinds of steps towards it can be considered morally permissible and politically legitimate. But the input from people of “political wisdom” remains necessary, be they other researchers, activists, officials, intellectuals, or all of the above.

## Three Desiderata for Good Theories of Change

What makes a *good* nonideal theory? Rawls argues that nonideal theory should look for “policies and courses of action that are morally permissible and politically possible as well as likely to be effective” (Rawls, 2001, 89). This is usually read as a list of three criteria: moral permissibility, political possibility, and likely effectiveness (Garner, 2013; Milligan, 2015). Notice, however, that there is also a fourth criterion, namely that nonideal theory should look for “policies and courses of action” at all. This criterion marks a crucial difference between ideal and nonideal theory. Ideal theory should supply a snapshot description of a just society in the form of principles of justice. Nonideal theory should describe a *process of transition*. Although it could do so in a vague and unhelpful way (e.g., “just make all regimes comply!”; “just let prisoners of war live!”), a *good* nonideal theory will describe this process of transition in a way that is helpful in guiding the actions of agents in the real world—it will propose policies and courses of action.

The desideratum of providing “policies and courses of action” can be reformulated as a set of desiderata for TOCs in nonideal theory:

First, a TOC should determine who needs to do what in order for ideal justice to be achieved in the long run. Only a TOC that is sufficiently clear on this point can be action-guiding. Rawls’s foreign relations example determines that *the governments of well-ordered peoples* are to *set up intergovernmental institutions, exert symbolic pressure using these institutions*, and *reinforce symbolic pressure with economic pressure*. While this does not yet fix what any particular person should do, it focuses the attention on specific, crucial agents, the governments of well-ordered peoples. What other agents should do—such as citizens, elected officials, political parties, and interest groups—derives from what these crucial agents should do.

Second, a TOC should determine who should be expected to be unable or unwilling to comply and how they should be enabled or compelled to comply. If it does not, it fails to address the basic challenge of nonideal theory, namely, deliberate noncompliance and unfavourable circumstances. Rawls’s foreign relations example supposes that *non-well-ordered regimes* should be expected to *deliberately refuse compliance out of self-interest*, and that they should be moved to comply with principles of justice *by a combination of symbolic and economic pressure*. His slavery example supposes that it is *the ruling elites* who should be expected to refuse compliance with justice and that they should be moved to comply *by appeal to economic self-interest*, since keeping prisoners of war as slaves is more profitable than killing them, and exchanging them for their own prisoners of war is yet more profitable.

Third, a TOC should provide compelling reasons for the choice and order of its intermediate steps. Usually, these reasons will have to make reference to the institutions within or around which change must be achieved. For example, the reason why introducing slavery for prisoners of war is the wisest move is that the ruling elites are unwilling to exchange prisoners of war right away, but they are willing to introduce slavery, and will in time become willing to exchange prisoners of war. In the foreign relations example, the reason why intergovernmental institutions should be established is that this is the most feasible way to create a forum where symbolic pressure can be exerted upon non-well-ordered regimes and measures to exert economic pressure can be coordinated.

To be clear, a TOC that suffices all three desiderata succeeds in providing “policies and courses of action”. Whether those policies and courses of action are “morally permissible and politically possible as well as likely to be effective” (Rawls, 2001, 89) is then a question philosophers should debate. Thus, sufficiently specific TOCs are the beginning of a discussion, not the end. In the next section, let me explain why I believe that animal ethics has not seen this beginning yet.

## Animal Ethics and Theories of Change

To repeat, the charge of lack of realism in animal ethics typically targets theorists like Singer (2002), Regan (2004), and Francione (1995). It comes in at least two distinct variants. The first variant asserts that the vision of justice these approaches present is too demanding for human beings even under favourable circumstances (Garner, 2013; Cochrane et al., 2018; Kasperbauer, 2018). Thus, they fail to provide a good ideal theory, which should present a *realistic* utopia.

The second variant claims that these approaches to animal ethics are not sufficiently responsive to the fact that relevant numbers of people currently refuse to comply with their demands (Garner, 2013; Cochrane et al., 2018). The problem the critics see here is that views that ignore overwhelming unwillingness to comply are not helpful in actually effecting change (Cochrane et al., 2018, 267).

A questionable assumption of both variants is that theorists like Singer (2002), Regan (2004), and Francione (1995) should offer either an ideal or a nonideal account of justice between human beings and animals. On their own terms, they appear to be after specific principles of justice or morality, such as the Principle of Equal Consideration of Interests, the Respect Principle, or the principle that animals should be legal persons, not property. But one can argue for such principles and explore their demands without claiming that they provide a *comprehensive* description of a just human-animal coexistence, let alone a plan for how to achieve it. And if they offer only a partial picture of a just coexistence, it is not obvious that this partial picture by itself needs to be responsive to real-world restrictions. This search for certain principles of justice or morality, which does not claim to establish a *comprehensive* account of a just coexistence, we might call *foundational* animal ethics as opposed to *ideal* animal ethics.

Both versions of the charge however capture that, for better or worse, foundational animal ethics is not directly action-guiding. At best, it makes *pro tanto* prescriptions, but not prescriptions *all things considered*. To be action-guiding, it needs to be expanded or embedded into a comprehensive ideal theory of justice and then supplemented with a nonideal theory. To this limited extent, the charge of lack of realism in animal ethics is fair.

The point of advancing the charge, however, is usually to motivate some constructive contribution to nonideal theorizing. As we will shortly see, contributions have discussed the validity of various approaches to animal advocacy, suggested how policy goals could be made more achievable, and highlighted tactical ideas for the animal protection movement. As imaginative and helpful as all these contributions are, they cannot provide a sufficiently specific TOC in nonideal animal ethics. Thus, even at their most “pragmatic”, animal ethicists have not yet provided what well-intentioned decisionmakers and activists need.

What we have seen in the debate can be summarized in three broad categories of nonideal theorizing:

*Critique.* Much nonideal theorizing in animal ethics focuses on critique, in the sense that they object that a given strategic approach lacks promise in real-world circumstances. Examples abound, from the critique of the sudden abolition of animal use (Garner, 2013) to that of incremental welfare reform (Francione, 1995), that of strategies of individual behaviour change (Best, 2014; Calarco, 2016; Donaldson & Kymlicka, 2016; Delon, 2018) and of moral persuasion (Best, 2014; Tuider, 2016; Leenaert, 2017), all the way to the critique of liberal democratic reform (Cavalieri, 2016) and of pacifism (Best, 2014).

The point of such critiques is typically to show that the approaches they target cannot succeed because they are overdemanding or because they misconstrue the nature of injustices committed against animals. But while such critiques are important, they are not sufficient to describe a path to justice. One can be very precise about what *not* to do while only gesturing vaguely towards what to do. Critique is often clear and facts-responsive, but its positive action-guidance is no more straightforward than that of foundational animal ethics. What nonideal theorists have suggested in positive terms usually falls into one of the remaining two categories:

*Compromise*. Although animal ethicists are usually aware of Rawls’s conception of ideal and nonideal theory, they do not always appreciate the difference between Rawlsian nonideal theory and mere political compromise, in the sense of a settlement between the ideal and the current state of things. Some straightforwardly define nonideal theory as balancing or compromising between ideal theory and the status quo (Garner, 2013, 90; Eskens, 2021, 266f.). Some also characterize nonideal theory as ideal theory’s politically achievable counterpart (Milligan, 2015, 166; Schmitz, 2016, 40; Cochrane et al., 2018, 267).

The problem with nonideal theory as compromising theory is that it misses the essentially transitional character that makes nonideal theory action-guiding. By itself, the description of a compromise does not tell us who needs to do what, against whose opposition, and for what reasons, so that *either* the compromise *or* the ultimate goal of ideal justice is achieved. Apart from lacking essential information, mere compromising also cannot inspire the ingenious, path-dependent TOCs we may sometimes need. For example, mere compromising between killing all prisoners of war and exchanging all prisoners of war would not lead us to temporarily introduce slavery. If anything, it would lead us to fight for the exchange of *some* prisoners of war rather than all—which, as Rawls stipulates the case, would be *less* achievable.

Consider the most prominent example of explicitly nonideal animal ethics in the literature, Garner’s account of animal rights (Garner, 2013). Garner argues that an interest-based theory of rights called the “enhanced sentience position” is a good ideal theory. According to this view, animals with an interest in continued life deserve a right to life, though this right is only as strong as the interest that underpins it. Assuming that humans have a stronger interest in continued life than nonhumans, their right to life is stronger (Garner, 2013, 133). Still, granting animals this comparatively lightweight right to life would require major revisions to economies and administrations. Hence, Garner advises that we should first opt for compliance with the less ambitious “sentience position”: Animals have an interest-based right not to be made to suffer, but they have no right to life (Garner, 2013, 123). The sentience position is intended to be a compromise between the enhanced sentience position and the status quo (Garner, 2013, 137). In accordance with the sentience position, societies may use free-range husbandry, animal-based in-vitro technologies, and animal disenhancement that removes the capacity to suffer (Garner, 2013, 136). These technologies involve using animals as means to human ends in ways that require killing them, but not in ways that require inflicting suffering.

Interesting as it is, the sentience position is not sufficient to provide a sufficiently specific TOC. It does not tell us who needs to do what in order to make any particular society comply with its principles (desideratum 1). It vaguely tells us something about who will be unable or unwilling to comply—a “public” no further specified (Garner, 2013, 138). But it does not tell us how this opposition should be addressed (desideratum 2). Garner does provide reasons to think that the sentience position is a necessary halfway point between the status quo and ideal justice (desideratum 3). They are that rights claims in general can be a powerful tool for political movements, but animals’ right to life is much more contested than their right not to be made to suffer (Garner, 2013, 137). However, the fact that one right is more popular with the people than another—or *less un*popular—is no compelling reason to take it as a strategic reference point. After all, both rights are far from being viable policy proposals in any of today’s liberal democracies. Granted, certain highly specific and unusual forms of animal use would remain permissible, but they are not the forms of animal use in which powerful industries are invested and which they will fight to uphold. So although Garner gestures towards reasons for why his compromise would be an important transitional step, he omits too much information for the TOC to be compelling.

The situation is similar with Ladwig’s (2020) proposal that the introduction of animal rights should begin by qualifying the kind of reasons that can legally justify harm to animals. Only *morally significant* human interests must be weighed against the morally significant interests of animals (Ladwig, 2020, 381). This system is halfway between the status quo, which treats trivial (e.g. culinary, economic) human interests as justificatory, and Ladwig’s ideal theory, which grants animals rights that trump mere interests. Ingenious though this proposal is, it does not specify who should do what, against whose opposition, and in what steps, to achieve either the compromise or the ultimate goal.

In sum, the problem with nonideal theory as compromising theory is that it does not provide agent-centered TOCs, and the TOCs we can try to extrapolate from a compromise alone are too indeterminate to meet the desiderata. Therefore, while compromises might form important parts of TOCs, they are not sufficient by themselves.

*Tactics*. Finally, the most common kind of nonideal theorizing in animal ethics focuses on tactics. By a tactic, I mean a general pattern of goal-directed action that can be applied to a range of particular situations, as opposed to a strategy, which would identify a particular course of action in a particular context. For example, various authors argue that animal advocates should use human-centered arguments about health and the environment to get people to switch to a plant-based diet (Fetissenko, 2011; Tuider, 2016; Sebo & Singer, 2018). Cooney (2011) and Leenaert (2017) focus on persuasion tactics, Joy (2008) on organizing tactics, and Kasperbauer (2018, 173–196) on nudging and other psychological interventions. Cavalieri, among other things, proposes a “long march through the institutions” and the “penetration into social casemates like the publishing system, magistracy, and especially universities” (Cavalieri, 2016, 30). Donaldson and Kymlicka call for alliance building with other progressive causes (Donaldson & Kymlicka, 2016, 84), Ladwig for civil disobedience (2020), Best for militant direct action (Best, 2014).

Morally and politically evaluating these tactical ideas is worthwhile and helpful. But just like a toolbox cannot replace a building manual, a set of tactics cannot replace a TOC. Many different agents could employ tactical ideas in many different contexts. What we would need, in addition to tactics, is an account of who in particular should use which of them, against whose opposition, and in what interim steps.

More detailed and process-oriented tactical advice can be found in the work of legal and political scholars drawing lessons from past policy change (e.g. Anderson, 2011; Burger, 2013; Bloomfield, 2015). The advice in this literature often comes remarkably close to providing a sufficiently specific TOC, except that is not determinately linked to an ideal theory of justice.

For example, Anderson (2011) considers the history of the abolition of legalized child labour in Great Britain. He concludes that policy change towards stronger protection for the powerless is possible even at the expense of powerful interests if it follows four stages: (1) Industrialization leads to increased competition, which leads to a deterioration of conditions for the powerless; (2) Credible public figures and interest groups promote a new ethic that objects to these conditions, building symbolic pressure for reform, though policies must be economically feasible and success may depend on external trigger events (e.g. a catastrophe that raises public awareness); (3) Interest groups and officials push for domestic legislation, consumers voice their wishes through boycotts, and attorneys push for highly visible litigation both to change the law and further increase public pressure; (4) Upon legislative success, activists take measures to prevent cooptation, dilution, and circumvention of policies.

While this may pass for a sufficiently specific TOC, it is not a TOC *in nonideal animal ethics*. It does not answer the question how ideal justice may be achieved under real-world circumstances, only how specific policies for the protection of the powerless can be achieved. This is helpful to decisionmakers and activists who already have preconceived policies, but not to those looking for policies and courses of action to pursue in the first place.

Perhaps the closest we have come to seeing a sufficiently specific TOC in animal ethics is in Donaldson and Kymlicka's work (2016). First, the authors appeal to their comprehensive ideal vision of a just human-animal coexistence, *Zoopolis* (Donaldson and Kymlicka 2011). According to this ideal theory, animals and human beings ought to be considered rights bearers, with different animals having different sets of rights depending on their relation to human-animal communities. While all animals should have a suite of universal basic rights, domesticated animals should additionally have an analogue of citizenship, with which come animal citizenship rights (e.g. the rights to medical care, sharing public space, or political representation, Donaldson and Kymlicka 2011, 126, 142, 153). Liminal animals like urban foxes should have an analogue of a residence permit, but not full animal citizenship rights. Wild animals should be treated like citizens of another sovereign state, in which humans should only intervene in emergency cases where that sovereignty is threatened.

Second, Donaldson and Kymlicka appeal to Anderson’s four-stage model of policy change (Donaldson & Kymlicka, 2016, 82) and a range of other tactical ideas (Donaldson & Kymlicka, 2016, 84–104). So what exactly shall we do here and now, against whose opposition, and why? Unfortunately, Donaldson and Kymlicka stop just short of giving a unified account of what needs to happen for a *Zoopolis* regime to be implemented in any particular nation state. Their emphasis lies on adding to the “collective imaginary” of the animal protection movement (Donaldson & Kymlicka, 2016, 77). Important and valuable as this is, it does not fully address the situation of the well-meaning decisionmaker or activist who looks to animal ethics for guidance. Furthermore, part of a political movement’s collective imaginary consists in coherent visions of change—think of the various theories of revolution and reform in traditions of the left. In sum, even though valuable work has been done in providing tactical ideas and linking them up with comprehensive visions of a just human-animal coexistence, animal ethics is still facing a dearth of sufficiently specific TOCs.

## Imagining Transition in Animal Ethics

Presumably, one reason why animal ethicists have avoided theorizing transition is that it requires some bold speculation and there are many “unknown unknowns”. TOCs are likely to be naive, in the sense of making implausible or uncertain assumptions, particularly about future events and developments. Nonideal theories based on naive TOCs are unlikely to pass the Rawlsian desiderata of suggesting policies and courses of action that are *politically possible* and *likely to be effective*. But nonideal theory that refuses to commit to a specific TOC fares no better. I suggest, instead, that we should embrace naiveté for a start. A naive TOC is better than none, and it can be improved upon.

Let me sketch a TOC to convey what I have in mind. It answers the question: Assuming that *Zoopolis* (Donaldson and Kymlicka 2011) presents an adequate ideal theory of a just human-animal coexistence, how could a regime along its lines come about in real-world circumstances? Since *Zoopolis* is a vision for a liberal democratic nation state, and because transition to *Zoopolis* will require manoeuvering within and around the institutions of some given state, a helpful TOC should be state-specific. Let me focus on the state with whose institutions I am most familiar, Switzerland.

### Planning Backwards: The Upkeep Phase

It can be helpful to narrate transition in reverse order, beginning with the result. As Anderson (2011), Burger (2013), and Donaldson and Kymlicka (2016) point out, all policy success is liable to relapse and reaction. So the end point of the TOC is not the *introduction* of a *Zoopolis* regime in Switzerland, but its successful *upkeep*. The most important condition for this, apart from institutional feasibility, is that there is a general consensus among the Swiss people that this regime is just and legitimate. Public institutions can strengthen this consensus by performing a public culture of memory and conscious confrontation of past injustices, embodied in museums, monuments, public admissions of guilt, and “truth commissions” (Scotton 2017). But the necessary consensus can only arise and prevail against the backdrop of an economy that is nearly completely *Zoopolis*-compatible before the regime’s introduction, so that economic interests will not be strong enough to agitate against, revert, or dilute its policies, or subvert them by extralegal means (e.g. creation of black markets, circumvention by imports). The state, too, will need to have been *Zoopolis*-compatible for some time, fulfilling similar functions for the protection and participation of animals. While the state is the crucial agent in the upkeep phase of the *Zoopolis* regime, various individuals and institutions must help enable and push it to accomplish its tasks. In particular, citizens and animal protection groups will have to constantly keep public pressure up.

### The Introduction Phase

As there will already need to be a public consensus about the justice and feasibility of the *Zoopolis* regime, its introduction should not be imagined as a revolution. Its eventual formalization would be largely an administrative move, perhaps with some substantive improvements in the treatment of animals sprinkled in. It would resemble the introduction of the first Swiss Animal Welfare Act in 1978, which likewise brought certain improvements for animals, but was largely a synthesis of functionally similar legislation that had previously existed at the level of cantons (see Häsler, 2010).

Even if the introduction of the *Zoopolis* regime will not represent a drastic shift in animal protection and their political participation, the administrative switch will require significant revisions to the Swiss constitution and to legislation at all administrative levels. Previous political structures that take animal interests into account, which might vary by canton and be partially informal, will need to be replaced by a new, uniform system of interest representation in the *Zoopolis* regime (perhaps a third chamber of parliament comprised of animal representatives).

Another fundamental revision is the introduction of a new type of legal personhood. Swiss law distinguishes between natural persons (human beings) and juridical persons (e.g. corporations). Animals could be considered either a new subclass of natural persons or their own type of “animal person” (Stucki, 2016). In either case, they will be considered bearers of legal rights, but not legal duties. Basic animal rights and different sets of group-dependent animal rights will also need to be formalized.

Making fundamental constitutional revisions is comparably straightforward in Switzerland, as they can be proposed at any time by the chambers of parliament or by popular initiatives, both of which trigger a binding popular vote. The crucial agent at this stage is therefore the Swiss people, who need to pass the constitutional changes at the ballot box. Given that the economy will already be largely *Zoopolis*-compatible and the state will already have similar tasks and structures, the debate should be imagined to revolve around issues such as the efficiency and security of the new system, only marginally about substantive improvements in the protection and political participation of animals.

### The Preparatory Phase

Obviously, there are enormous obstacles standing in the way of the introduction phase: the Swiss people’s inability and deliberate unwillingness to agree to measures that would functionally resemble those of a *Zoopolis* regime. The reasons are presumably myriad, but we can gauge some of the most important factors: economic, cultural, and scientific reliance on using animals in ways that are *Zoopolis*-incompatible, lack of preparedness to consider animals (e.g. in the construction of buildings and roads, conservation, landscape management, energy production, city planning…), and unwillingness to enable animals to participate in political decision making. Hence, there first needs to come a long phase of preparatory politics. Its aim is to make the Swiss economy independent from relying on using animals in ways that violate their future rights and to make the state’s institutions consider and include animals in all areas that affect them (see Wild, 2019; Ladwig, 2020). In other words, the aim is to make the economy and state “*Zoopolis*-compatible” ahead of time, step by step.

The preparatory phase should be imagined as a long-term project, spanning several human generations. Public opinion—people’s beliefs, values, sense of entitlement—is not immobile, but it is inert, slow to follow changes in material conditions of life that are themselves piecemeal and sluggish. Guided by this conception, the preparatory phase advances from minor to major changes in life conditions as public opinion shifts, forming a positive feedback loop (see also Fesenfeld et al., 2021).

The crucial agent here is the Swiss state, particularly the confederation (the highest administrative unit). For it is the confederation who funds a great deal of research and development, distributes agricultural and other subsidies, establishes advice centres, runs public information campaigns, and promotes Swiss consumer industries against foreign competition. When it comes to animal experimentation, the confederation already has a mandate to encourage the development, recognition, and use of animal-friendly alternative methods (Art. 22 AWA) and follows suit by funding a “3R competence centre” and major research projects. These and other measures the confederation could use, without the use of coercion or any major interference in the economy and citizen’s private lives, to enable and compel producers, investors, and consumers to gradually move towards an increasingly *Zoopolis*-compatible economy. The problem is that the confederation is not yet willing to use its means to this end. So other agents, particularly political parties, interest groups, scholars, and citizens need to identify *Zoopolis*-conducive policies that enjoy majority support and compel the confederation to adopt them.

For example, when it comes to the meat industry, the state will need to be moved to fund research and development of *Zoopolis*-compatible alternatives to meat, be they plant-based or truly synthetic; to use subsidies to encourage producers to switch from husbandry to producing plant proteins and other agricultural products; to provide consultation services for producers seeking to switch; to launch information campaigns on responsible plant-based nutrition and report transparently about the meat industry’s impact on animals and the environment; and to cut its current subsidies to pro-meat advertising, taking simultaneous measures to discourage meat imports. Eventually, the adoption of an official phase-out plan for meat may become feasible, but only after a considerable shift in public opinion towards the very end of the preparatory phase.

The state itself will not become *Zoopolis*-compatible by these measures alone. Rather, legislation will have to be gradually improved to approximate the structure of *Zoopolis*. Animal protection legislation will have to be expanded and strengthened, and innovation experiments in animal political participation will have to be launched. Towards later stages of the preparatory phase, as legislative procedures become more animal-friendly themselves, there will be an acceleration towards *Zoopolis*.

However, we should generally expect legislative transition to follow economic transition, not vice versa (contrary to “New Welfarism”, Francione, 1998). Just as a ban on gassing male chicks in the egg industry will only follow the introduction of an economically viable technology for in-ovo sexing, the various prescriptions and prohibitions that will make the state *Zoopolis*-compatible will usually follow changes in what is economically viable. However, the state can *make* more *Zoopolis*-like arrangements economically viable by diverting subsidies, funding research and development, and so on. Because the confederation does not currently operate on the view that the state and the economy should become *Zoopolis*-compatible, the onus is on citizens, elected officials, political parties, and interest groups to develop, draft, and submit policies to these ends.

Opposition will be powerful and democratic majorities will have to be fought for at every step along the way. However, notice that the piecemeal measures envisioned here do not run counter to majority values, and many are even compatible with powerful economic interests. For instance, installing consultation services, increasing subsidies for plant proteins, and publicly funding research and development of alternatives to meat do not directly threaten majority food preferences or the interests of the meat industry. Presumably, opposition to such policies will primarily come from libertarian circles opposed to increasing the confederation’s budget. A well-organized alliance of left and green parties in conjunction with animal protection, environmental, and small agricultural interest groups should suffice to overcome this opposition.

The situation is more difficult with other policies—such as diverting subsidies away from animal products and cutting public funding to pro-meat advertising—that run counter to powerful economic interests. However, notice that the opposition to progressive policies will not always be unified. One camp will presumably push a nationalist, protectionist line, arguing that Swiss animal industries must be protected from foreign competition in the interest of food security. Another camp will be more concerned with decreasing the confederation’s budget and reducing state interference in the economy. Clever policy proposals will divide-and-conquer these two camps. However, it will take a strong alliance of citizens, elected officials, parties, and interest groups to successfully campaign against the opposition.

In sum, the preparatory phase of politics revolves around the state turning the economy and itself *Zoopolis*-compatible by noncoercive means. The primary emphasis is not on improving animal welfare legislation, but on removing obstacles for a *Zoopolis*-compatible society. Over time, as the conditions of life shift, so does public opinion. The crucial agent in this phase is the state, and a progressive alliance should enable and compel it to make the right moves, overcoming the opposition of industries and nationalist or libertarian circles.

### The Buildup Phase

The general obstacle for the preparatory phase is that the crucial alliances still need to be built. In some cases, left and green political parties play the role of deliberate noncompliers, showing little interest in allying with animal advocates. The crucial agents who will need to act are largely animal protection groups, since successful alliance-building requires collectives, not just individuals. On the one hand, animal protection groups will need to increase their capacity to communicate, mobilize, research, and develop policy, in order to become more capable and attractive allies. This also prepares them for the later phases of the TOC, where they will be needed as partners in policy development and raising public pressure. Animal protection groups might continue some of their previous activities—campaigning, fundraising, networking, and so on. What this TOC suggests, however, is that organizations’ focus in this phase should be on building political capacity and establishing alliances much more than on the actual impact of campaigns (contrary to Effective Altruism). Another way to increase political capacity is to directly spend resources on movement building, for instance on networking, education, skill and knowledge sharing.

On the other hand, animal protection groups will need to help political parties and elected officials to recognize the importance of animal issues. This requires that animal protection groups present their own cause in a way that is at least compatible with other justice struggles (Donaldson & Kymlicka, 2016). But a major part of the problem is material rather than ideal: Most parties do not have committees or working groups focusing on animal issues, do not publish policy papers nor have sections of the party programme dedicated to them. There are few people advocating for animals within these structures, and few arenas for them to advocate in. This can change however, as the recently introduced animal protection working groups of two Swiss youth parties show (JG, 2020; JGLP, 2021). Animal protection groups can help to establish structures and encourage their members to participate in them. At least in parties where *Zoopolis*-compatible policies generally align with established environmental and social concerns, the creation of such structures should not be—and have in fact not been—met with significant opposition.

At this point, finally, the TOC becomes action-guiding for an individual citizen in Switzerland today: Join a collective effort, particularly on the part of animal protection groups and political parties, to build political power and establish alliances for policy change towards a *Zoopolis*-compatible Swiss economy and state.

This advice may sound trivial, but a TOC needs to be specific, not surprising. In fact, if its guidance were too far removed from what real-world agents are already motivated to do, that would be a source of non-compliance the TOC has not addressed. At the same time, one should not overstate the triviality of the TOC stated above. Typically, the Swiss animal protection movement focuses on achieving immediate policy success, persuading people to go vegan, or promoting animal welfare programmes. The TOC suggests that, during the first phase, the primary purpose of these activities should be the buildup of political power by means of structures, alliances, networks, knowledge, and other resources. Policy change should become a primary goal only later on, and economic policies should be particularly high on the agenda. So the TOC considerably reorients the animal protection movement’s strategic outlook.

The bulk of nonideal work for philosophers only *begins* here, namely, debating in what respects the proposed policies and courses of action are politically possible, morally permissible, and likely to be effective. One could argue, for instance, that the TOC envisioned above is overly optimistic in assuming that public opinion will shift with material conditions of life. Or that a TOC would be more democratically legitimate if it began by giving animals a fair say in political decision-making.^1^ This is the kind of debate I hope to facilitate by offering a specific TOC.

## Conclusion

I have argued that the charge of a lack of “realism” in animal ethics is partially fair, insofar as foundational theory is not directly action-guiding. To be action-guiding, foundational animal ethics needs to be set in the context of a comprehensive ideal theory of a just human-animal coexistence, and then supplemented with a sufficiently specific TOC. Such a TOC should determine (1) who needs to do what in order for ideal justice to be achieved in the long run, (2) who should be expected to refuse compliance and how they should be moved to comply, and (3) why specific intermediate steps are called for given the institutions at issue. The dominant types of nonideal theorizing in animal ethics so far—critique, compromise, and tactics—do not provide sufficiently specific TOCs by these standards. To convey a sense of what a TOC could look like, I have sketched a TOC that describes a path from a particular nonideal context today, Switzerland, to a particular ideal theory of justice, *Zoopolis*. The TOC consists of a buildup phase, a preparatory phase, an introduction phase, and an upkeep phase, determining crucial agents, crucial opposition, and reasons for the intermediate steps along the way. Although the TOC is still naive in certain respects, it is determinate enough to suffice the three desiderata and provides a template for how animal ethics can be made action-guiding under real-world circumstances.

## Footnotes

1 I thank an anonymous reviewer for this point. My worry is that this is infeasible until the later stages of the preparatory phase, but I would welcome the articulation of a “representation-first” TOC.
